# Citrullination in health and disease: From physiological function to gene regulation

**DOI:** 10.1016/j.gendis.2024.101355

**Published:** 2024-06-22

**Authors:** Xiaoya Zhang, Guiqiu Xie, Lang Rao, Chaoguang Tian

**Affiliations:** a National Technology Innovation Center of Synthetic Biology, Key Laboratory of Engineering Biology for Low–Carbon Manufacturing, Tianjin Institute of Industrial Biotechnology, Chinese Academy of Sciences, Tianjin 300308, China; b School of Pharmacy, Jilin University, Changchun 130012, China

**Keywords:** Citrullination, Deimination, Histone, Peptidyl arginine deiminase, Therapeutic interventions, Transcriptional control

## Abstract

Protein citrullination involves the deimination of arginine or methylarginine residues in peptide chains to form citrulline by peptidyl arginine deiminases. This process is an important protein post-translational modification that affects molecular structure and function of various proteins, including histones. In recent years, protein citrullination has attracted widespread attention for its influence on gene transcription. Studies on the impact of protein citrullination modification on chromatin structure remodeling and the establishment of gene regulatory networks have made rapid progress. In this review, we briefly summarize the physiological functions of protein citrullination modification. Specifically, we comprehensively outline the latest progress in the study of the role of protein citrullination modification in gene transcription regulation, focusing on the interaction of protein citrullination with other post-translational modifications.

## Introduction

Post-translational modification (PTM) refers to the process of chemical modification of proteins after synthesis. This process involves enzymatic addition and removal of chemical moieties such as acetylation, methylation, phosphorylation, ubiquitination, and ADP-ribosylation of proteins.^1^^,^^2^ These modifications regulate intracellular biological processes by affecting protein structure, function, and interactions with other biomolecules. Citrullination is a less-studied PTM involving the catalytic conversion of positively charged arginine or methylarginine residues into neutrally charged citrulline, which alters protein–protein and protein-nucleic acid interactions.^3^^,^^4^ In addition, citrullination influences the activity of histones and non-histone proteins such as keratin, filaggrin, myelin basic protein, vimentin, actin, and enolase. These proteins play crucial roles in regulating gene expression, chromatin structure, cell signaling, and other physiological processes. Though the function of protein citrullination has been studied for various proteins, its roles and regulatory activity remain incompletely understood.^5^^,^^6^

Citrullination is primarily carried out by the peptidyl arginine deiminases (PAD). These enzymes convert arginine residues into citrulline residues, thereby altering the charge and function of the protein. Citrulline residues were first discovered in the early 1960s in polypeptide hydrolysates from the inner root sheath and medullary cells of hair.^7^ Five highly conserved, calcium ion-dependent citrullinases (PAD1-4 and 6) have been identified in human. PAD1-4 exhibit catalytic activity, whereas PAD6 lacks this activity due to mutations in the active site.^8^, ^9^, ^10^, ^11^, ^12^ PAD1, PAD2, and PAD4 can enter the nucleus, although only PAD4 possesses a nuclear localization signal. Interestingly. PAD2 has also been observed in the nucleus, despite lacking a nuclear localization signal.

Although all the human PAD isozymes are highly conserved with over 50% sequence similarity, they differ in tissue distribution and substrate preference. Interestingly, PAD produced by *Porphyromonas gingivalis* (PPAD) is a unique enzyme. Unlike human PADs, PPAD does not require calcium for catalysis and is directly associated with inflammation, tissue destruction, and the development of oral diseases.^13^^,^^14^ Research on PPAD offers critical insights into the connection between the biological role of citrullination modification and the pathogenesis of associated diseases.^15^

This review seeks to provide a summary of the current knowledge regarding protein citrullination modification. Specifically, we intend to emphasize the recent advancements of citrullination in gene transcription regulation research.

## Citrullination and peptidyl arginine deiminases

**Figure 1:**
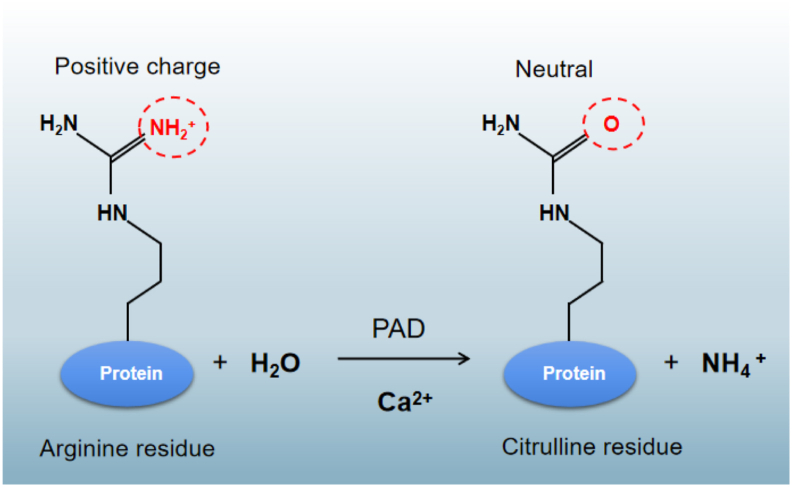
The reaction mechanism of protein arginine citrullination is catalyzed by PAD.

Citrullination of proteins, also called deimination, involves the conversion of an arginine residue into a citrulline residue by the removal of an imino group, resulting in the loss of a positive charge and a small molecular weight change (+0.98 Da) (Fig. 1).^3^^,^^7^ This positive charge loss affects protein structure, function and interaction with other biomolecules.^16^ Citrullination also has a substantial impact on the pH of the amino acid side chain, reducing the isoelectric point of the modified arginine from 11.41 to 5.91, thereby affecting the acidity of the protein, the formation of hydrogen bonds, and electrostatic interactions between amino acids.^17^^,^^18^ No enzymatically driven process has been discovered that catalyzes the reverse conversion of citrulline to arginine. Thus, protein citrullination is an irreversible modification, which differs, for example, from phosphorylation. This irreversible reaction enables longer-lasting signal transmission and functional regulation of biochemical process. Understanding the mechanisms and functions of is essential to reveal its significance in cells and biological systems. Moreover, identifying methods to reverse or remove protein citrullination is a crucial area of ongoing research.^18^^,^^19^

Calcium plays a crucial role in regulating human citrullinases. The sensitivity of PAD to calcium ion regulation is due to its unique structural and functional characteristics, as calcium ions help PAD fold into the correct protein conformation and maintaining stable structural. ^4^Under normal physiological conditions, PADs usually exhibit low basal activity. However, its activity is regulated by various factors, including inflammatory signals, intracellular pH, which can lead to corresponding changes in calcium ion concentration. Studies have shown that the intracellular calcium ion concentration needs to be in the micromolar range to activate PADs.^20^^,^^21^ In-depth research on the regulation mechanism of calcium ions will facilitate a better understanding of the PADs and provide new solutions and targets for treating related diseases.^22^

## Physiological functions of citrullination and pathological conditions of abnormal citrullination

**Figure 2:**
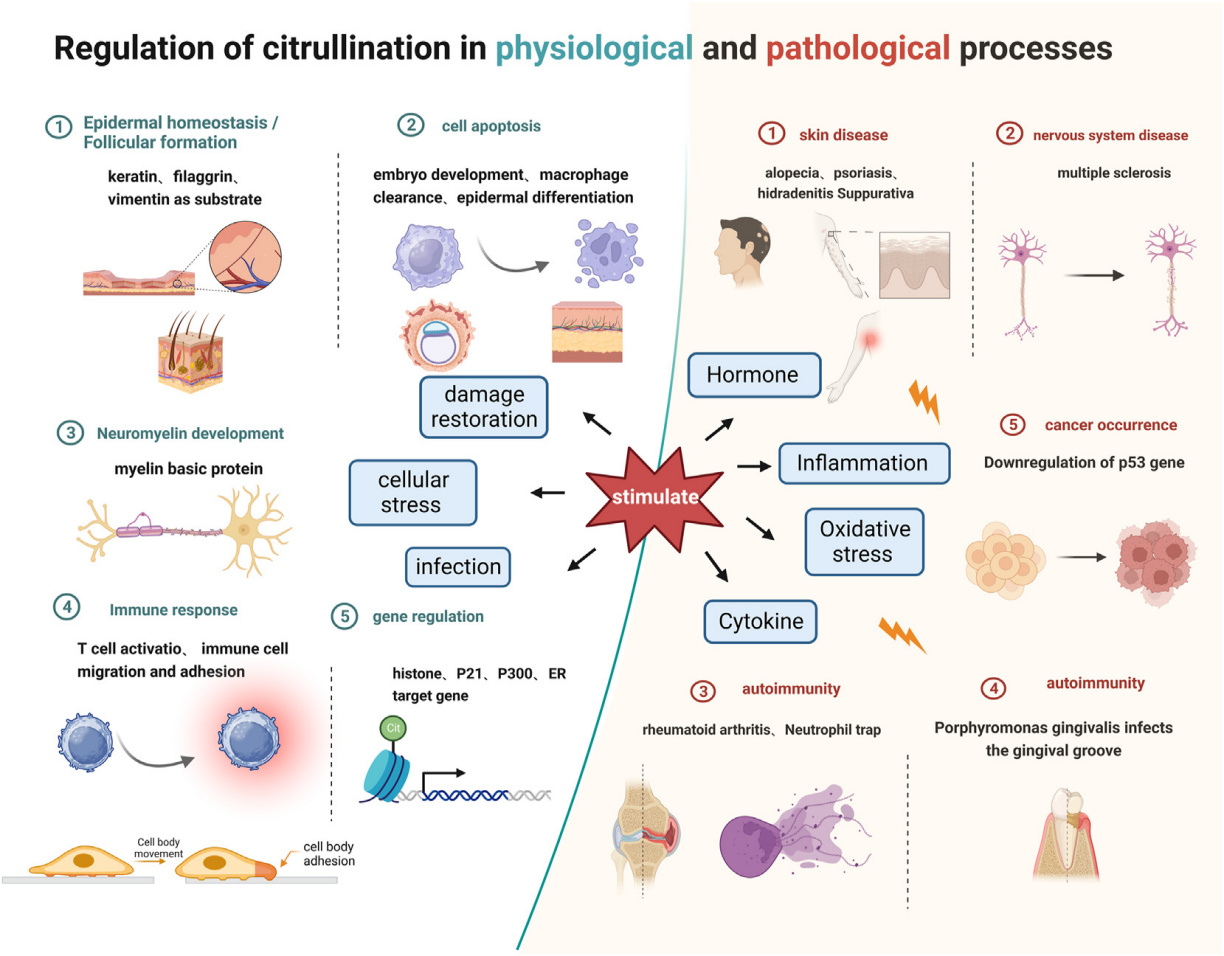
**Protein citrullination in physiological and pathological processes.** Under physiological conditions, PAD is activated in specific cellular environments such as cell proliferation and differentiation, cellular stress and damage repair, hormonal regulation and immune modulation. Depending on its expression in different tissues, PAD regulates skin homeostasis and formation and participates in cell apoptosis, neuronal myelination development, the immune response and gene regulation. An imbalance in one of these activation signals or PAD dysfunction, can lead to abnormal levels of citrullination, which may contribute to the development of conditions such as cicatricial alopecia, dermatological disorders, multiple sclerosis, rheumatoid arthritis and cancer.

Extensive experimental evidence has unveiled distinct expression patterns of each PAD gene across various cell types, tissue types, cell differentiation stages, and under diverse physiological or pathological conditions (Fig. 2).^11^^,^^23^^,^^24^ Further investigations into the regulation of PAD gene expression are expected to yield a more comprehensive and precise understanding of the functions and regulatory mechanisms of the PAD enzyme family in different biological processes.

PAD1 is mainly expressed in the epidermis and endometrium, and is also expressed in hair follicles and keratinocytes, where it is closely associated with epidermal keratinization,^8^^,^^25^^,^^26^a process in which several epidermal proteins, including filaggrin and the K10 and K26 isoforms of cytokeratin, are citrullinated to form a protective matrix in the skin.^27^ Furthermore, during citrullination of cytokeratin, the charge loss can dissociate the cytokeratin-filaggrin complex.^42^^;^^43^As filaggrin degrades, it can produce the amino acids of natural moisturizing factor, which play an essential role in skin moisturization.^28^^,^^29^

PAD2 is the most widely expressed citrullinase in tissues. It is distributed in various organs, including secretory glands, central nervous system (CNS), uterus, spleen, and kidney, as well as in some immune cells such as macrophages.^30^ Substrates of PAD2 include myelin basic protein, vimentin, actin, enolase and histones. In macrophages, PAD2 induces citrullination of vimentin, rendering it unable to perform its normal function of stabilizing the position of organelles in the cytoplasm, subsequently leading to structural abnormalities and apoptosis. PAD2 is also involved in epigenetic regulation.^47^^,^^48^ Estradiol stimulation induces PAD2 to be recruited to the target promoter region through hormone receptor estrogen receptor α (ER). PAD2 citrullinated H3R26, resulting in local chromatin decondensation and transcriptional activation.^31^ PAD2 is also an androgen-suppressing protein whose expression is increased in prostate cancer. The expression of PAD2 is crucial for the survival, cell cycle development, and proliferation of prostate cancer cells.^32^, ^33^, ^34^, ^35^ Therefore, PAD2-mediated histone citrullination may become a potential target for the treatment of prostate cancer.^32^

PAD3 is primarily located in the skin epidermis and hair follicles and is also found in peripheral nerves. Its substrate proteins mainly include filaggrin, trichohyalin, and enolase.^36^ PAD3 can regulate the apoptosis process mediated by apoptosis-inducing factor and participate in the formation of cytoskeletal organization through the action of citrulline.^37^ Trichohyalin hairpin protein (THH) is present in the medulla of hair follicles and inner root sheaths and is the main target of PAD3. The citrullination of THH mediated by PAD3 enhances the function of root sheath cells in hair follicles and induce hair growth.^38^ In addition, vitamin D can increase the expression of PAD3 in cultured keratinocytes, and this derivative has potential benefits in the treatment of psoriasis.^39^^,^^40^

PAD4 is widely distributed in immune cells, such as macrophages, neutrophils, and eosinophils, and is also present in the brain, uterus, bone marrow, and joint tissues.^41^^,^^42^ Compared to the other PAD family members, PAD4 has more catalytic substrates. Its substrates including transcription factor SOX4, Histone acetyltransferase p300, P21, nucleophosmin, Lamin C, Ribosomal protein S2, DNMT3A, and histones.^43^, ^44^, ^45^ In neutrophils, it plays an important role in chromatin decondensation, gene regulation and the formation of neutrophil extracellular traps (NET)s through citrullination of histones H1 and H3.^46^ In addition, PAD4 also involves in the pathogenesis of Rheumatoid arthritis (RA), PAD4-mediated citrullination of collagen can reduce the adhesion of synovial fibroblasts and mesenchymal stem cells and change the pathogenesis of RA.^47^ PAD4 is overexpressed in a majority of tumor tissues, including osteosarcoma, colon adenocarcinoma. In cancer cells, PAD4 catalyzed histone citrullination coupled with HDAC2 catalyzed deacetylation represses p53 target gene expression.^48^ Citrulline formation is also inextricably linked to autoimmunity. For example, studies have shown that PAD4 mediates the citrullination of histone H3 and promotes the formation of NETs. And inflammation-induced NETs have been shown to awaken dormant cancer cells through proteolytic remodeling of laminin.^49^^,^^50^

PAD6 is mainly expressed in the ovary and plays a crucial role in oocyte growth, fertilization and early embryonic development.^51^ It has been detected in early embryos, testes and thymus.^52^^;^^53^Citrulline formation also appears to be important for female fertility, with mice lacking PAD6 being infertile due to defects in cytoskeletal sheet formation in early embryos. Consequently, PAD6 has been proposed as a target for contraceptive drugs.^45^

PPAD is a non-human PAD originally discovered from *Porphyromonas gingivalis* which was the primary bacterial located in the human oral cavity. It uses fibrinogen, α-enolase, and epidermal growth factor as citrullination substrates.^9^^,^^13^^,^^15^^,^^54^ PPAD rapidly degrades bacterial and human host proteins such as fibrinogen and alpha-enolase. PPAD released by *P. gingivalis* can diffuse into the host's connective tissue and citrullinate epidermal growth factor, preventing its recognition by the epithelium. This mechanism delays the local healing process and destroys the local protective epithelial cell-periodontal tissue barrier, playing an important role in the development of periodontitis.^50^^,^^51^

PADs also serve as diagnostic markers and therapeutic targets for multiple diseases, playing a significant role in human health. Therefore, research on PADs as diagnostic markers and therapeutic targets holds vital clinical significance, providing potential opportunities for developing new treatment strategies and drugs. Table 1 summarizes the latest findings on the physiological functions of PAD isoforms and their identified molecular targets and protein substrates. When one of the activating signals is imbalanced (hormones, immune responses, oxidative stress or cytokines) or PAD activity is dysregulated, abnormal levels of citrullination may contribute to the development of diseases such as neurodegenerative diseases, muscle diseases, Cancer development, and autoimmune diseases (Fig. 2).^44^^,^^55^, ^56^, ^57^, ^58^, ^59^, ^60^

**Table 1:** PAD enzymes: expression, citrullination substrates and physiological functions.

PAD isotype	Expression	Citrullinated substrate	Physiological function
PAD1	Epidermis uterus, hair follicle, keratinocyte^25^^,^^27^^,^^38^^,^^40^^,^^61^	Keratin, filaggrin	Epidermal tissue keratosis, skin differentiation, epithelial-mesenchymal transformation^20^^,^^36^^,^^61^
PAD2	Secretory glands, brain, uterus, spleen, kidney, pancreas, skeletal muscle, macrophage, oligodendrocytes, yolk sac (white blood cells), skin, peripheral nerves, immune cells, inner ear^56^^,^^62^, ^63^, ^64^, ^65^	Myelin basic protein, vimentin, actin, enolase, Histone H3(R2, R8, R17, R26), Histone H4 (R3)	Cell differentiation and myelination, NET formation, female reproduction, gene regulation, tumorigenesis^31^^,^^36^^,^^43^^,^^62^
PAD3	Epidermis, hair follicles, peripheral nerves^12^^,^^66^	Fibroin, Trichomonas protein, enolase, S100A3	Lower stratum corneum epidermal homeostasis, skin differentiation, hair follicle formation^20^^,^^36^^,^^67^
PAD4	Immune cells, brain, uterus, joints, bone marrow, cancerous tissue^68^, ^69^, ^70^	ING4, p300, P21, nuclear phosphorus, laminin C, RPS2, DNMT3A, Histone H1(R54), Histone H2A(R3), Histone H3(R2, R8, R17, R26), Histone H4 (R3, R23)	Tumorigenesis, NET formation (including in COVID-19 patients), gene regulation, apoptosis, antimicrobial natural immunity^20^^,^^36^^,^^71^^,^^72^
PAD6	Ovaries, eggs, early embryos, testes, thymus^73^	α- tubulin	Regulates oocyte skeleton sheet formation and female fertility, regulates microtubule function, and reproductive system^25^^,^^36^^,^^73^
PPAD	buccal cavity^15^	Fibrinogen, alpha-enolase, epidermal growth factor	Periodontitis occurs^13^^,^^15^^,^^74^^,^^75^

## Role of citrullination in gene regulation

### Histone citrullination in gene regulation

**Figure 3:**
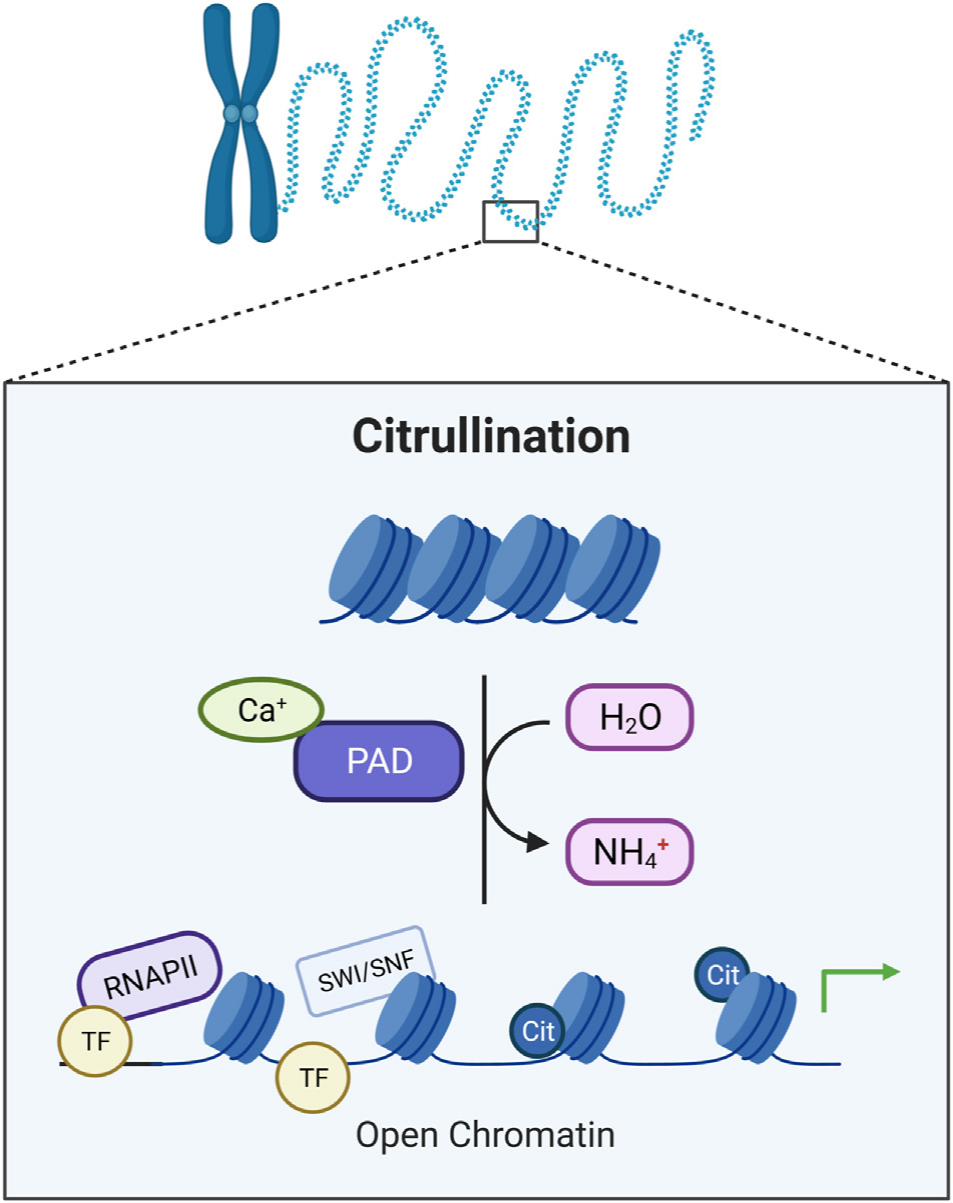
**Chromatin opening through PAD-mediated citrullination of histone tails.** Citrullination of histone tails removes positive charges, thus enhancing the repulsive force between histones and DNA. This PTM facilitates the access of DNA-binding proteins to DNA. The binding and recruitment of SWI/SNF complex remodeling factors promote the binding of sequence-specific factors, enabling more effective access of RNA Polymerase II and transcription factors to transcribe DNA into mRNA.

In recent years, citrullination has garnered significant attention as a potential regulatory factor in gene transcription. Studies have revealed an interplay between citrullination and transcription factor activity, the remodeling of chromatin structure and the establishment of gene regulatory networks(Fig. 3).^76^, ^77^, ^78^

Citrullination of the N-terminal tail of histones is related to transcriptional regulation, and this modification could be found on all four types of histones H1, H2A, H2B, H3, and H4^79^(Table 2). Numerous studies have unequivocally demonstrated the pivotal functions of PAD2 and PAD4 in facilitating gene activation. Upon exposure to β-estradiol in ERα-positive cells, researchers have observed the recruitment of PAD2 to target gene promoters, instigating citrullination of histone H3 arginine 26 (H3R26) on chromatin. Consequently, this process induces local chromatin opening and triggers transcriptional activation.^31^ Furthermore, the direct impact of citrullination by PAD2 on the upregulation of interleukin-6 has been established.^76^ This discovery further substantiates the involvement of PAD2 in regulating inflammation and immune responses. Conversely, citrullination by PAD2 facilitates normal differentiation of oligodendrocytes, myelination and motor function.^80^ Additionally, PAD4 plays a pivotal role in governing the binding of regulatory elements for key stem cell genes, thereby activating their expression and unveiling the contribution of citrullination in pluripotency regulation. These findings further reinforce the indispensable role of PADs in transcriptional regulation and offer crucial insights for understanding the functions and mechanisms underlying citrullination modifications.^81^

**Table 2:** Distinct citrullination sites of arginine residues on histones.

Histone	Citrullination site(s)
H1	54
H2A	3, 11, 77, 81, 88
H2B	29, 31, 33
H3	2, 8, 17, 26, 42, 49, 52, 117
H4	3, 17, 19, 23, 35, 36, 39, 40, 45, 94

Citrullination enzymes can counterbalance the actions of histone arginine methyltransferases during the process of citrullinating histone arginine residues, thereby effectively suppressing gene expression. Within the CCCTC-binding factor (CTCF) promoter, PAD4 inhibits the PRMT4-mediated H3R17me2a modification by actively antagonizing arginine methylation and forming interactions with T-cell acute lymphocytic leukemia protein 1 (TAL1). This inhibitory effect by PAD4 leads to a decrease in CTCF gene expression levels.^82^ This discovery highlights the pivotal role of PAD4 in regulating estrogen signaling, as it disrupts histone methylation to finely modulate gene expression levels.^26^ Thus, citrullination enzymes inhibit gene expression by counteracting PTMs generated by histone arginine methyltransferases.

In conclusion, citrullination at the N-terminal tails of histones closely correlates with transcriptional regulation. PAD2 and PAD4 play pivotal roles in gene activation, inflammation regulation, oligodendrocyte differentiation and the regulation of genes in stem cells. Delving deeper into the intricacies of the mechanisms and functionalities underlying citrullination will provide molecular details governing gene regulation. Moreover, such investigations should identify novel targets and strategies for treating associated diseases.^83^^,^^84^

### Non-histone citrullination in gene regulation

In addition to modifying histones, PADs regulate mRNA transcription by directly affecting transcription factors and epigenetic regulators, thereby acting as transcription cofactors. The most current research findings focus on PAD4.^65^^,^^85^^,^^86^

Erythroblast transformation-specific-like protein-1 (Elk-1) is a critical transcription factor promotes functional changes in cells. Studies have revealed that PAD4 interacts with Elk-1 on the c-Fos promoter, and upon EGF stimulation, PAD4 catalytic activity promotes phosphorylation of Elk-1, acetylation of histone H4 and transcriptional activation of c-Fos.^77^ Furthermore, PAD4 affects P300 activity through citrullination by removing a site within the methylated by CARM1. This citrullination-mediated regulation by PAD4 influences the bimolecular interaction between P300 and glucocorticoid receptor-interacting protein 1.^87^ Additionally, through citrullination, PAD4 modulates the functionality of DNA methyltransferase 3A (DNMT3A). Studies have demonstrated that elevated levels of PAD4 expression enhance the expression and stability of DNMT3A, which is potentially achieved by regulating DNMT3A degradation sensitivity.^88^

In inflammatory cells, PAD4-mediated citrullination of the E2F-1 transcription factor impedes E2F-1-driven cell apoptosis and enhances the role of E2F-1 in inflammatory responses.^89^ Moreover, PAD4 directly citrullinates NF-kB p65, boosting its signaling transduction and reinforcing the interaction between p65 and importin α3, a protein responsible for the nuclear translocation of p65, thereby promoting its nuclear localization.^90^ Additionally, PAD4 exerts regulatory effects on hematopoiesis and leukemia gene expression. Researchers have discovered the interaction between PAD4 and T-cell acute lymphocytic Tal1, acting as a coactivator to activate the expression of the IL-6 cytokine family signal transducer (IL6ST) gene. Within the Tal1/PAD4 target gene IL6ST, PAD4 counteracts the inhibitory H3R2me2a mark triggered by PRMT6, enhancing the active H3K4me3 mark, thereby increasing the expression of IL6ST. This activity suggests that PAD4 interacts with the central hematopoietic transcription factor Tal1, regulating gene expression in erythroid and leukemic cell lines.^82^

PAD4 plays a role in regulating pro-apoptotic genes. The transcription factor p53 recruits PAD4 to the OSGIN1 promoter, facilitating the expression of this gene.^37^ Additionally, PAD4 can be recruited by p53 to the p21 gene to suppress p21 transcription. Studies have revealed a negative correlation between histone H3R17 methylation and H3 citrullination, accompanied by a reduction of RNA Pol II at the promoter region. Inhibition or depletion of PAD4 enhances the expression of a subset of p53 target genes, including cyclin dependent kinase inhibitor 1A, leading to cell cycle arrest and apoptosis.^91^

In conclusion, the role of PADs in gene regulation is far more complicated than previously understood, and further exploration of the regulatory roles played by other enzymes in the PAD family is needed. PADs exert regulatory control of gene expression through multiple pathways, and citrullination of proteins by PADs has significant regulatory effects on PTMs of other proteins. These research findings provide a fresh perspective for a deeper understanding of the functions and regulatory mechanisms of PADs in gene regulatory networks.

## Crosstalk between citrullination and other protein post translational modifications

Citrullination of proteins, similar to other PTMs, can significantly affect the formation of neighboring modifications like methylation and acetylation. This interplay, known as ‘crosstalk,’ can subsequently impact the recruitment of specific ‘reader’ proteins, thereby influencing protein–protein interactions and biological processes including gene expression. The interaction of histone modifications is a growing research area in epigenetic regulation, with studies demonstrating the interplay of two to three modifications in altering gene expression.^92^, ^93^, ^94^, ^95^, ^96^

### Crosstalk with methylation

**Figure 4:**
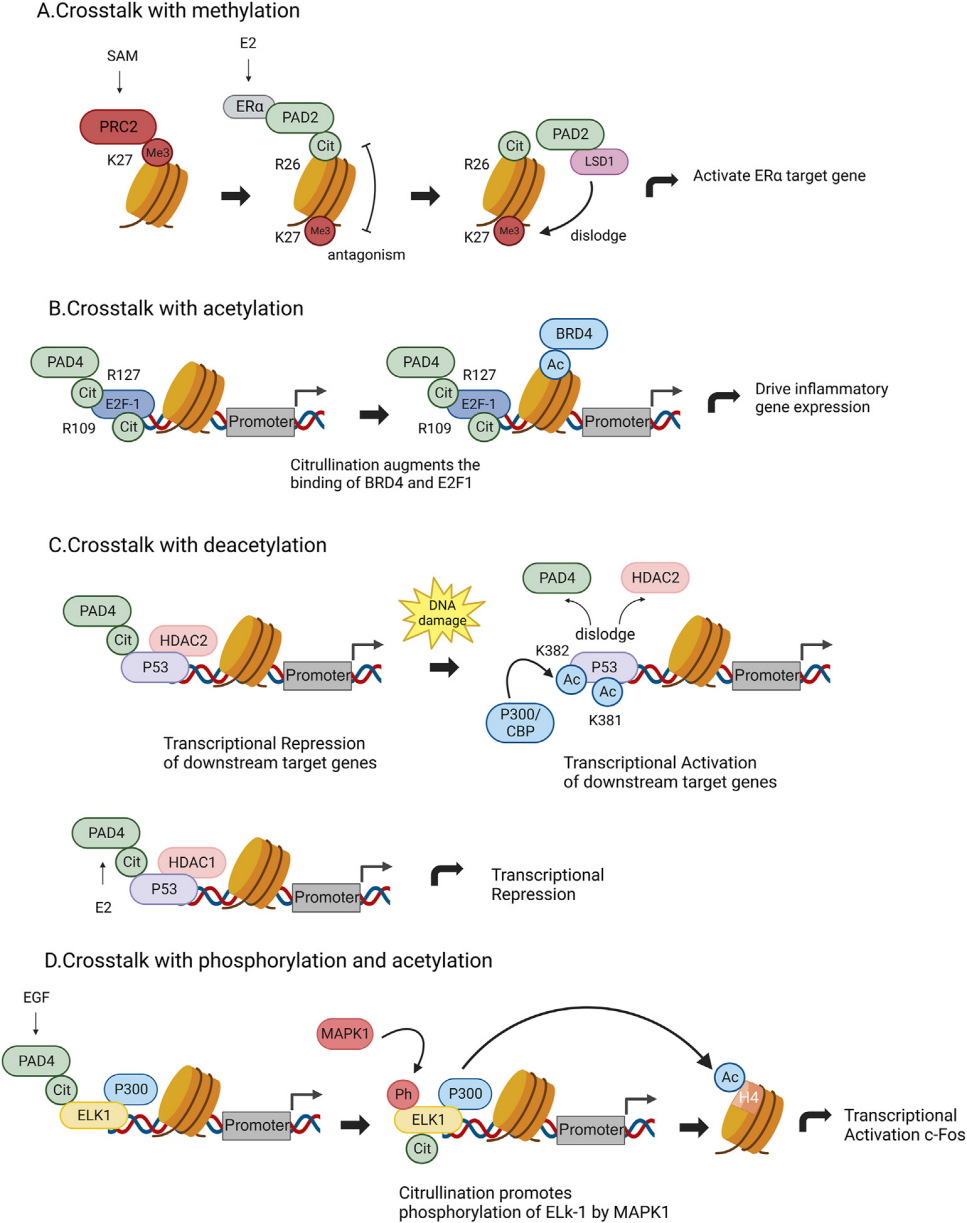
**Crosstalk diagram between citrullination and other modifications.** (A) PAD2 citrullination and PRC2 complex antagonism and crosstalk with LSD1 regulate ERα gene expression. SAM: S-adenosyl methionine (cofactor); PRC2: polycomb repressive complex 2 (methyltransferase activity); E2: 17-β-estradiol; LSD1: lysine-specific demethylase. (B) PAD4-mediated crosstalk between citrulline and acetylation. PAD4 promotes the recruitment of E2F-1 to cytokine target gene promoters, where E2F-1 interacts with BRD4 to drive inflammatory gene expression. (C) PAD4 and HDAC1/2 collaborate to regulate the expression of target genes. HDAC2 and PAD4 interact with p53 through distinct structural domains, leading to the downregulation of gene expression at p53 target gene promoters. Following DNA damage, PAD4 and HDAC2 dissociate from p53 target gene promoters, and recruitment of P300/CBP for acetylation results in transcriptional activation. (D) The PAD4-mediated c-Fos activation illustrates the crosstalk facilitated by the co-factor, Elk-1. On the c-Fos promoter, Elk-1 forms a complex with p300. Upon stimulation by EGF, the catalytic activity of PAD4 promotes the phosphorylation of Elk-1 by mitogen-activated protein kinase 1 (MAPK1). This phosphorylation event enhances the interaction between Elk-1 and p300, leading to acetylation of histone H4 and transcriptional activation of the c-Fos gene.

The most obvious form of crosstalk between citrullination and other PTMs is observed with arginine methylation. Recent research showed a strong negative crosstalk between EZH2-mediated H3K27me3 methylation and PAD2-mediated H3R26 citrullination. Specifically, citrullination of H3R26 completely blocks methylation at the K27 site, whereas methylation of H3K27 reduces the rate of citrullination at the R26 site. Further investigations found that under E2 stimulation, ERα recruits PAD2 to chromatin to citrullinate H3R26, whereas PAD2 recruits lysine demethylase 1A(LSD1) for demethylation of K27, thereby activating transcription of ERα target genes (Fig. 4A).^97^ Another example of citrulline–methylation crosstalk on histones was caused by PAD4. PAD4 function as an epigenetic regulator by affecting histone 3R2me2a methylation. In nature, transcription factor Tal1 form a protein complex with the PAD4, and mediate its recruitment to genes involved in leucocyte differentiation such as IL6ST and CCCTC-binding factor (CTCF). On promotor of IL6S, PAD4 acts as a coactivator by counteracting the repressive H3R2me2a mark created by PRMT6. This action enhances the active H3K4me3 mark, thereby promoting the expression of IL6ST. Conversely, on the CTCF gene promoter, PAD4 acts as an inhibitory factor. It counteracts the activating mark H3R17me2a mediated by PRMT4, resulting in low expression of the CTCF gene.^82^

### Crosstalk with acetylation

The interplay between histone citrullination and acetylation enables gene transcription repression. As discussed above,In granulocyte cells, PAD4 citrullinates E2F-1 at multiple sites, including Arg109 and Arg127. This citrullination enhances the binding of the bromodomain reader BRD4 to an acetylated domain in E2F-1. As both PAD4 and BRD4 are present with E2F-1 located on cytokine gene promoters, PAD4 caused citrullination regulating E2F-1-mediated cell apoptosis and boosting E2F-1's role in inflammatory responses (Fig. 4B).^89^^,^^98^

Studies have shown that histone deacetylase 2 (HDAC2) and PAD4 interact with different domains of p53. p53 is an active DNA and chromatin-binding protein that selectively regulates the expression of its target genes by directing specific co-factors to different binding sites. On downstream target gene promoters of p53, such as p21, GADD45 and PUMA, p53 interacts with HDAC2 and PAD4 to regulate gene expression.^99^^,^^100^ After DNA damage, PAD4 and HDAC2 dissociate from several p53 target gene promoters, accompanied by an increased histone Lys acetylation and Arg methylation of these promoters. In this process, P300, through recruitment by P300-mediated upregulation mechanisms, participates in the regulation of gene expression (Fig. 4C).^101^

Additionally, studies have revealed a dynamic interaction between PAD4 and histone deacetylase 1 (HDAC1). PAD4 and HDAC1 bind to the pS2 promoter, and knockdown experiments of HDAC1 showed a decrease in H3 citrullination and an increase in histone arginine methylation, indicating that PAD4 and HDAC1 synergistically establish an inhibitory chromatin environment at the pS2 promoter.^102^

### Crosstalk with phosphorylation

ELK-1 is activated in response to MAPK/ERK signalling, and phosphorylation of Elk-1 leads to enhanced association with the p300 acetyltransferase, with subsequent induction of acetylation of target genes and enhanced gene activation. PAD4 has been demonstrated to interact and citrullinate Elk-1. Upon stimulation by EGF, PAD4 facilitates ERK2-mediated phosphorylation of Elk-1 This phosphorylation enhances the interaction between Elk-1 and p300,^85^ thereby mediating the acetylation of histone H4 and transcriptional activation of the target gene c-Fos(Fig. 4D).^77^ These findings define a novel role for PAD4 as a co-activator of transcription factors. In conclusion, as an important histone modification, citrullination not only interferes with other PTM states but also regulates gene expression by modulating the activity and function of transcription factors.

## PADs as therapeutic target for disease treatment

The enzymatic process of protein deimination, facilitated by PAD enzymes, has been implicated in the pathophysiology of diverse diseases, including autoimmune disorders, cancers, and neurodegenerative conditions.^45^^,^^103^, ^104^, ^105^, ^106^, ^107^ Consequently, the inhibition of PAD enzymes has emerged as a promising therapeutic strategy.

## Reversible inhibitors

A variety of broad-spectrum reversible inhibitors targeting PAD enzymes have been identified, such as Cl-amidine, BB-Cl-amidine, F-amidine, treptomycin, minocycline, paclitaxel、and chlortetracycline.^79^^,^^108^^,^^109^ Cl-amidine and BB-Cl-amidine inhibit PAD activity by forming covalent bonds with the active site cysteine, thereby preventing arginine to citrulline conversion.^79^^,^^110^ In contrast,F-amidine targets an auxiliary domain of the PAD enzyme, modulating its enzymatic function differently.^111^ Notably, paclitaxel, a mitotic inhibitor employed in cancer therapy, inhibits PAD2 by interfering with microtubule polymerization, showcasing a novel inhibition mechanism.^111^ This class of inhibitors allows for the reversible suppression of PAD activity, providing a temporary therapeutic effect that can be reversed upon inhibitor withdrawal.

## Irreversible inhibitors

Irreversible PAD enzyme inhibitors bind irreversibly to the PAD enzyme. This binding permanently destroys the enzyme's activity. Such inhibitors generally have higher affinity and longer duration of action and can therefore be used at lower concentrations. Irreversible PAD enzyme inhibitors include Cl-amidine analogues, 2,3-dichloro-1,4-naphthoquinone (DCNQ), Chloroacetamidine, N-α-benzoyl-N5-(2-chloro-1-iminoethyl)-l- ornithine amide (Cl-ornithine).^79^^,^^112^^,^^113^ DCNQ is an irreversible PAD enzyme inhibitor that can form a covalent bond with the cysteine residue of PAD enzyme, thereby permanently inhibiting PAD enzyme activity.^114^^,^^115^ Chloroacetamidine will form an amide bond with the histidine residue in the PAD enzyme thereby permanently destroying the histidine residue in the active site of the PAD enzyme and preventing the PAD enzyme from catalyzing its substrate.^116^ It should be noted that irreversible PAD enzyme inhibitors have higher affinity and longer action time, but may also have irreversible effects on other proteins, leading to side effects. Therefore, caution is required when using irreversible PAD enzyme inhibitors.

PAD inhibitors can inhibit the activity of PAD enzymes through different mechanisms, regulating gene expression and post-transcriptional modifications by regulating protein deimination. These regulatory effects may help in the treatment of various diseases.

## Conclusions and future perspectives

Understanding the specific regulatory mechanism of PAD-mediated citrullination, including the biochemical properties of citrullination enzyme and its impact on protein modification, will help reveal the role of this modification process in cell function and disease pathophysiology. In addition, the synergistic effect of citrullinase and other protein modification residues or proteins in gene regulation allows us to use multiple selective inhibitors synergistically, which can bring great inspiration to the treatment of diseases. Currently, researchers are working to build more specific and powerful tools to manipulate the PAD enzymes and accurately detect its potential role in gene regulation. The development of these tools will help us better understand the functions and regulatory mechanisms of PAD enzymes in cellular processes.

## Conflicts of interest

The authors declare that there is no conflict of interest.

## Funding

This work was supported by the Tianjin Synthetic Biotechnology Innovation Capacity Improvement Project (Grant No. TSBICIP-CXRC-048).
